# Supplementary material for: Effects of improved on-farm crop storage on perceived stress and perceived coping in pregnant women—Evidence from a cluster-randomized controlled trial in Kenya
Source: PLoS One. 2023 Jul 13;18(7):e0288446. doi: 10.1371/journal.pone.0288446 (PMC10343033; doi:10.1371/journal.pone.0288446)
Supplement: S1 Table — (DOCX) [file pone.0288446.s001.docx]

**S1 Table. Translation of the short form of the Perceived Stress Scale (PSS-4).**

|  | **Original version - English** | **Final translation to Swahili** |
| --- | --- | --- |
| PSS-4 Intro | The next questions ask you about your feelings during the last 30 days. Reply 1 to continue. | Maswali yanayofuata yanauliza kuhusu hisia zako katika kipindi cha siku 30 zilizopita. Jibu 1 Kuendelea. |
| PSS1 Perceived Stress | In the last 30 days, how often have you felt that you were unable to control important things in life? 1)never 2)almost never 3)sometimes 4)often 5)very often (Reply 1-5) | Siku 30 zilizopita, mara ngapi umeshindwa kuzuia mambo muhimu katika maisha? 1)Sijawahi 2)Nadra 3)Mara nyingine 4)Mara nyingi 5)Mara nyingi sana (Jibu 1-5) |
| PSS2 Perceived Coping | In the last 30 days, how often have you felt confident about your ability to handle personal problems? 1)never 2)almost never 3)sometimes 4)often 5)very often (Reply 1-5) | Siku 30 zilizopita, mara ngapi umehisi una uwezo wa kudhibiti shida zako? 1)Sijawahi 2)Nadra 3)Mara nyingine 4)Mara nyingi 5)Mara nyingi sana (Jibu 1-5) |
| PSS3 Perceived Coping | In the last 30 days, how often have you felt things were going your way? 1)never 2)almost never 3)sometimes 4)often 5)very often (Reply 1-5) | Siku 30 zilizopita, mara ngapi umehisi mambo yanakuendea unavyotarajia? 1)Sijawahi 2)Nadra 3)Mara nyingine 4)Mara nyingi 5)Mara nyingi sana (Jibu 1-5) |
| PSS4 Perceived Stress | In the last 30 days, how often have you felt difficulties were piling up so high that you couldn’t overcome them? 1)never 2)almost never 3)sometimes 4)often 5)very often (Reply 1-5) | Siku 30 zilizopita, mara ngapi umehisi shida nyingi unashindwa kuzidhibiti? 1)Sijawahi 2)Nadra 3)Mara nyingine 4)Mara nyingi 5)Mara nyingi sana (Jibu 1-5) |
