# Supplementary material for: Effects of improved on-farm crop storage on perceived stress and perceived coping in pregnant women—Evidence from a cluster-randomized controlled trial in Kenya
Source: PLoS One. 2023 Jul 13;18(7):e0288446. doi: 10.1371/journal.pone.0288446 (PMC10343033; doi:10.1371/journal.pone.0288446)
Supplement: S2 Table — (DOCX) [file pone.0288446.s002.docx]

**S2 Table. Cumulative variances of principal component analysis (PCA) for one-factor model and two-factor model.**

|  | Model | |
| --- | --- | --- |
| Month | One-Factor | Two-Factor |
| 1 | 0.39 | 0.67 |
| 2 | 0.40 | 0.72 |
| 3 | 0.42 | 0.72 |
| 4 | 0.44 | 0.74 |
| 5 | 0.43 | 0.74 |
| 6 | 0.43 | 0.73 |
| 7 | 0.44 | 0.75 |
| 8 | 0.45 | 0.75 |
| 9 | 0.41 | 0.72 |
| 10 | 0.40 | 0.72 |
| 11 | 0.42 | 0.73 |
| 12 | 0.47 | 0.75 |
| One-factor model: PSS1 + PSS2_r + PSS3_r + PSS4; two-factor model: PSS1 + PSS4 and PSS2_r + PSS3_r | | |
