# Supplementary material for: Effects of improved on-farm crop storage on perceived stress and perceived coping in pregnant women—Evidence from a cluster-randomized controlled trial in Kenya
Source: PLoS One. 2023 Jul 13;18(7):e0288446. doi: 10.1371/journal.pone.0288446 (PMC10343033; doi:10.1371/journal.pone.0288446)
Supplement: S3 Table — (DOCX) [file pone.0288446.s003.docx]

**S3 Table. Minimum and maximum factor loadings for one-factor model and two-factor model.**

|  | **Factor loadings** | |
| --- | --- | --- |
|  | One-Factor | Two-Factor |
| PSS1 | 0.60-0.77 | 0.79-0.89 |
| PSS2_r | 0.63-0.76 | 0.69-0.85 |
| PSS3_r | 0.30-0.63 | 0.86-0.89 |
| PSS4 | 0.55-0.75 | 0.82-0.89 |
| One-factor model: PSS1 + PSS2_r + PSS3_r + PSS4; two-factor model: PSS1 + PSS4 and PSS2_r + PSS3_r | | |
