# Supplementary material for: Effects of improved on-farm crop storage on perceived stress and perceived coping in pregnant women—Evidence from a cluster-randomized controlled trial in Kenya
Source: PLoS One. 2023 Jul 13;18(7):e0288446. doi: 10.1371/journal.pone.0288446 (PMC10343033; doi:10.1371/journal.pone.0288446)
Supplement: S4 Table — (DOCX) [file pone.0288446.s004.docx]

**S4 Table. Sample characteristics of baseline variables between treatment and control group.**

|  | Control N = 766 | | Treatment N = 825 | |  |
| --- | --- | --- | --- | --- | --- |
|  | Mean | SD | Mean | SD | *P* value |
| Household size* | 6.66 | 2.52 | 6.4 | 2.35 | 0.03 |
| Age of Household Head* | 45.21 | 12.78 | 44.14 | 12.35 | 0.09 |
|  | Percent % |  | Percent % |  | *P* value |
| Female-Headed Household* | 17.49 |  | 19.93 |  | 0.24 |
| Approximate Month of Birth |  |  |  |  |  |
| Don’t want to tell | 9.01 |  | 7.03 |  | 0.52 |
| Before Oct 2020 | 44.91 |  | 44.12 |  |  |
| Oct-Dec 2020 | 25.72 |  | 26.55 |  |  |
| Jan-Mar 2021 | 11.36 |  | 13.21 |  |  |
| Apr 2021 or later | 9.01 |  | 9.09 |  |  |

Baseline characteristics*, which were collected from January to March 2019 and approximate month of birth indicated by the pregnant women during their first completed survey are presented in the table. Parametric t-test was used for household size and age of household head. The other variables were measured using chi-square test. Data regarding household size, age of household head and female-headed household are missing for two participants of the treatment group (N = 823).
