# Supplementary material for: Effects of improved on-farm crop storage on perceived stress and perceived coping in pregnant women—Evidence from a cluster-randomized controlled trial in Kenya
Source: PLoS One. 2023 Jul 13;18(7):e0288446. doi: 10.1371/journal.pone.0288446 (PMC10343033; doi:10.1371/journal.pone.0288446)
Supplement: S5 Table — (DOCX) [file pone.0288446.s005.docx]

**S5 Table. Effects of improved on-farm storage on stress coping abilities during pregnancy.**

| Month | Control | Treatment | ITT | CI lo 95 | CI up 95 | t value | *P* value | *m/n/CG/TG* |
| --- | --- | --- | --- | --- | --- | --- | --- | --- |
| 1 | 4.38 | 4.23 | -0.15 | -0.46 | 0.16 | -0.973 | 0.331 | 36/635/294/341 |
| 2 | 4.63 | 4.24 | -0.39 | -0.69 | -0.06 | -2.445 | 0.014 | 36/677/319/358 |
| 3 | 4.52 | 4.26 | -0.26 | -0.56 | 0.05 | -1.675 | 0.094 | 36/604/274/330 |
| 4 | 4.47 | 4.38 | -0.09 | -0.42 | 0.23 | -0.566 | 0.571 | 36/633/301/332 |
| 5 | 4.58 | 4.33 | -0.25 | -0.58 | 0.08 | -1.508 | 0.131 | 36/539/252/287 |
| 6 | 4.47 | 3.96 | -0.51 | -0.82 | -0.19 | -3.173 | 0.002 | 36/504/229/275 |
| 7 | 4.43 | 4.13 | -0.30 | -0.68 | 0.07 | -1.547 | 0.122 | 36/496/232/264 |
| 8 | 4.19 | 4.07 | -0.12 | -0.60 | 0.31 | -0.577 | 0.564 | 36/446/203/243 |
| 9 | 4.53 | 3.99 | -0.54 | -0.90 | -0.14 | -2.795 | 0.005 | 35/410/196/214 |
| 10 | 4.09 | 3.96 | -0.13 | -0.53 | 0.26 | -0.635 | 0.525 | 36/396/182/214 |
| 11 | 4.13 | 4.26 | 0.14 | -0.28 | 0.53 | 0.665 | 0.506 | 36/375/176/199 |
| 12 | 4.11 | 3.99 | -0.11 | -0.52 | 0.32 | -0.535 | 0.593 | 35/369/159/210 |

The table presents the effects of improved on-farm storage on perceived coping during pregnancy, which is expressed as mean values on the PSS-4 items 2 and 3. The first column (Month) shows the number of months in which the PSS-4 was filled out. ITT = Intention-to-treat. Negative ITT values correspond to favorable outcomes. CI show 95% bootstrapped confidence intervals, lower (lo) and upper (up). *P* values based on non-parametric two-tailed t-tests. The bootstrap is based on 1000 replications. Sample sizes by number of pairs (m), total number of observations (n), and number of observations in control (CG) and treatment (TG) group are reported in the last column. Month 7 (October) = start of new storage period.
