# Supplementary material for: Effects of improved on-farm crop storage on perceived stress and perceived coping in pregnant women—Evidence from a cluster-randomized controlled trial in Kenya
Source: PLoS One. 2023 Jul 13;18(7):e0288446. doi: 10.1371/journal.pone.0288446 (PMC10343033; doi:10.1371/journal.pone.0288446)
Supplement: S6 Table — (DOCX) [file pone.0288446.s006.docx]

**S6 Table. Effects of improved on-farm storage on perceived stress during pregnancy.**

| Month | Control | Treatment | ITT | CI lo 95 | CI up 95 | t value | *P* value | *m/n/CG/TG* |
| --- | --- | --- | --- | --- | --- | --- | --- | --- |
| 1 | 3.71 | 4.01 | 0.30 | -0.08 | 0.64 | 1.624 | 0.104 | 36/635/294/341 |
| 2 | 3.74 | 4.12 | 0.39 | 0.05 | 0.71 | 2.353 | 0.019 | 36/677/319/358 |
| 3 | 3.64 | 4.16 | 0.52 | 0.17 | 0.87 | 2.918 | 0.004 | 36/604/274/330 |
| 4 | 4.08 | 4.03 | -0.06 | -0.41 | 0.28 | -0.324 | 0.746 | 36/633/301/332 |
| 5 | 3.59 | 4.07 | 0.49 | 0.15 | 0.85 | 2.697 | 0.007 | 36/539/252/287 |
| 6 | 3.75 | 4.11 | 0.36 | 0.04 | 0.71 | 2.084 | 0.037 | 36/504/229/275 |
| 7 | 3.63 | 3.90 | 0.27 | -0.11 | 0.68 | 1.350 | 0.177 | 36/496/232/264 |
| 8 | 4.24 | 4.15 | -0.09 | -0.47 | 0.32 | -0.448 | 0.654 | 36/446/203/243 |
| 9 | 3.92 | 4.35 | 0.44 | 0.10 | 0.80 | 2.474 | 0.013 | 35/410/196/214 |
| 10 | 4.24 | 3.92 | -0.33 | -0.71 | 0.04 | -1.718 | 0.086 | 36/396/182/214 |
| 11 | 4.21 | 3.89 | -0.32 | -0.74 | 0.06 | -1.554 | 0.120 | 36/375/176/199 |
| 12 | 3.94 | 4.08 | 0.14 | -0.31 | 0.64 | 0.573 | 0.567 | 35/369/159/210 |

The table presents the effects of improved on-farm storage on the prevalence of perceived stress of the Perceived Stress Scale (PSS) during pregnancy, which is expressed as mean values. The first column (Month) shows the number of months in which the PSS was filled out. Dates are middle days in each survey round (each round was open for four days). ITT = Intention-to-treat. Negative ITT values correspond to favorable outcomes. CI show 95% bootstrapped confidence intervals, lower (lo) and upper (up). *P* values based on non-parametric two-tailed t-tests. The bootstrap is based on 1000 replications. Sample sizes by number of pairs (m), total number of observations (n), and number of observations in control (CG) and treatment (TG) group are reported in the last column. Month 7 (October) = start of new storage period.
