# Supplementary material for: Effects of improved on-farm crop storage on perceived stress and perceived coping in pregnant women—Evidence from a cluster-randomized controlled trial in Kenya
Source: PLoS One. 2023 Jul 13;18(7):e0288446. doi: 10.1371/journal.pone.0288446 (PMC10343033; doi:10.1371/journal.pone.0288446)
Supplement: Attachment — Submitted filename: Manuscript_PLOS ONE - Reviewed.docx [file pone.0288446.s007.docx]

Full title:

**Effects of improved on-farm crop storage on perceived stress and perceived coping in pregnant women – Evidence from a cluster-randomized controlled trial in Kenya**

Short title:

**Effects of on-farm crop storage on perceived stress and coping in pregnant women**

Heike Eichenauer^1^, Matthias Huss^2,3^, Michael Brander^2,3^, Thomas Bernauer^2,3^, Ulrike Ehlert^1*^

^1^ Clinical Psychology and Psychotherapy, University of Zurich, Switzerland

^2^ Institute of Science, Technology and Policy (ISTP), ETH Zurich, Switzerland

^3^ Zurich Knowledge Center for Sustainable Development & Informatics and Sustainability Research Group, University of Zurich, Switzerland

^*^Corresponding author
E-mail address: [u.ehlert@psychologie.uzh.ch](mailto:u.ehlert@psychologie.uzh.ch) (UE)

**Abstract**

**Background**

Food insecurity can be harmful to pregnant women, as pregnancy is a challenging period with increased maternal nutritional requirements to ensure optimal fetal development and health of the mother. Whether food insecurity negatively affects maternal health may depend on how stressful pregnant women perceive this food insecurity to be and how strongly they believe they can cope with it. In Sub-Saharan Africa, pregnant women from smallholder households suffer from food insecurity due to post-harvest losses, i.e., loss of crops because of inadequate storage. An agricultural intervention that improves crop storage has been shown to reduce food insecurity. However, it remains to be determined whether this agricultural intervention has an additional positive effect on pregnant women’s perceived stress levels and coping abilities.

**Methods and Findings**

In a randomized controlled trial, SMS-based mobile phone surveys were conducted to assess the causal effect of a food security intervention (improved on-farm storage of maize) on perceived stress and coping in pregnant women from smallholder households. The significant results revealed that pregnant women from treatment households experienced more perceived stress but better perceived coping abilities compared to pregnant women from control households. Uncertainty due to lack of experience, this might have contributed to the higher perceived stress, as the women could not easily judge the benefits and risks of the new storage technology. However, the technology itself is a tangible resource which might have empowered the pregnant women to counteract the effects of post-harvest losses and therefore food insecurity.

**Conclusion**

Our study is the first to examine the effect of a new storage technology on perceived stress and coping in pregnant women from smallholder households in Sub-Saharan Africa. Future research should focus on how this technology impacts maternal mental health in a broader sense and whether biological mechanisms, such as epigenetics, may underlie this association.

**Introduction**

Adverse environmental conditions during pregnancy, such as food insecurity, can negatively impact the psychological and physiological health of women as well as the development of the fetus [1–3]. Food deprivation can be particularly harmful to pregnant women in low-income countries, as pregnancy is a very challenging period with increased maternal nutritional requirements to ensure optimal fetal development and health of the mother [4–6]. Research has shown that undernutrition during pregnancy is associated with an increased risk of mortality and morbidity for both mother and offspring [4,7]. Food insecurity can lead to adverse perinatal outcomes such as low birth weight and preterm birth [8] as well as pregnancy complications such as inadequate pregnancy weight gain, hypertension, preeclampsia, gestational diabetes [4,9], and mental disorders in the mother [8].

Whether food insecurity during pregnancy provokes negative maternal health outcomes can depend on how stressful pregnant women perceive this environmental condition to be. According to Lazarus and Folkman’s transactional stress theory [10,11], an individual is in constant exchange with the environment through two cognitive appraisal processes, thus influencing his/her well-being. First, the individual determines whether a particular environmental circumstance is irrelevant, positive, or stressful to him or her. The evaluation of the environment as stressful depends on whether it is perceived as harmful, threatening, or challenging (primary appraisal). The primary appraisal of a stressful environment is followed by a secondary appraisal, in which the individual determines whether he or she has adequate resources and coping strategies to deal with the environment. Harmful and threatening environments are often accompanied by feelings of uncontrollability over the situation and may thus trigger negative emotions [10–12] (for more detailed information, see this review [13]). With regard to food insecurity, numerous studies have reported a positive association between increased food insecurity and impaired mental health in African populations [14]. For instance, pregnant Ethiopian women living in food-insecure households were four times more likely to experience psychological distress than their food-secure counterparts [15]. Psychological distress is an important component of overall mental health and a risk factor for depression [16–18]. Researchers have demonstrated that food-insecure pregnant women in Africa are up to five times more likely to suffer from depression [19,20].

In Sub-Saharan Africa (SSA), food insecurity is particularly high among smallholder farming households, attributable to factors such as climate change-related extreme events and seasonality, which hamper agricultural production [21–24]. For example, a 10-day delay in the onset of the rainy season increases the risk of seasonal drought, thus affecting the food security status of smallholder farmers in SSA [25]. Furthermore, heavy rainfalls can prevent smallholder farmers from adequately drying their crops before storage, which can lead to increased moisture and thus mold growth during the storage period, rendering the crop inedible and therefore lost [26–28]. Post-harvest losses (PHL) refer to the amount of crop lost due to factors such as crop handling and crop storage [28], with the latter accounting for the majority of PHL [29,30]. Indeed, a meta-analysis revealed that on average, 25.6% of harvested maize in SSA is lost due to insufficient crop storage [30]. Moreover, the lean season, i.e. a few months after the harvest, when food stocks are depleted and the coming crops were yet to be harvested, poses the challenge of maintaining food security in smallholder farming households [24,31], which can be further exacerbated by PHL.

Agricultural interventions that focus on PHL, a well-known contributor to food insecurity, may be helpful to counteract the negative effects of food insecurity on maternal mental health and the intergenerational adverse health effects for the offspring. One method to improve crop storage is the distribution of hermetic storage bags instead of conventional polypropylene bags. Hermetic bags have been shown to improve crop storage in Tanzania and thus reduce PHL significantly [32]. A reduction in food insecurity due to hermetic storage bags was also demonstrated among smallholder farmers from the project described in the present study [33]. However, its effect on perceived stress and perceived coping (two components of the transactional stress theory) in pregnant women still needs to be elucidated.

In summary, on the correlation level, numerous studies have shown that food insecurity is associated with poorer mental health outcomes. We take this research forward by investigating whether a particular intervention to improve food security can have beneficial mental health outcomes. We hypothesized that pregnant women in the intervention households (treatment) would experience lower perceived stress and higher perceived coping as compared to pregnant women in the control households. Following the randomly allocated intervention, we collected data using monthly SMS surveys.

**Methods**

**Study setting**

The present RCT was conducted in Kakamega County, Western Kenya and included all 12 sub-counties of Kakamega and 59 out of 60 wards. Smallholder farming, and maize as the main staple food, are dominant in this Sub-Saharan region. The study area is affected by seasonality, with the lean season typically beginning around April or May and a secondary smaller harvest taking place around January or February [33]. A map of the study region and the smallholder farming groups’ locations can be found in S1 Fig.

**Research design and intervention**

This study was embedded in a matched-pair, cluster-randomization design. Pair-wise matching of the farmers was performed using baseline variables such as food security and mean market distance [34]. After pairing, farmers were randomly assigned to the treatment or control group using spatial clustering. This resulted in 62 experimental clusters with 285 farmer groups (5′444 smallholder households). More detailed study information can be found in Huss et al. [33]. Of these 5’444 smallholder households, a subsample of 1,591 pregnant women took part in the present study.

The treatment group received five hermetic storage bags of the brand “AgroZ” per household and standardized training in their usage. Each bag has the capacity to store 100kg of maize. The intervention was implemented from 3^rd^ to 15^th^ September 2019 by our local partner International Centre of Insect Physiology and Ecology (*icipe*), with the training session developed by some of the authors of this paper. The control group continued storing their maize as usual – mostly in traditionally used polypropylene bags.

**Outcome variable**

The RCT was designed to assess the effects of the intervention (hermetic storage bags) on food security and a variety of health outcomes [35]. The present study focused on perceived stress and perceived coping, measured by the short form of the Perceived Stress Scale (PSS-4). The PSS is based on the stress theory of Lazarus [10,12] and measures the degree to which an individual perceives his/her life as stressful as well as the individual’s perceived ability to cope with stress. The original version of the PSS consists of 14 items [36]; nowadays, a 10-item version is commonly used, and the scale has been further reduced to four items [37]. While some researchers found that the PSS-14, -10 and -4 capture two distinct psychological factors, *coping* and *stress* [38–43], others challenge this notion [44,45], particularly for the PSS-4 [46,47]. The PSS questionnaires have acceptable psychometric properties and have been validated in different countries (see review, [40]), as well as for pregnant women [48–50]. As the PSS-10 has been widely used to study Kenyan populations [51–57], it is reasonable to assume that the PSS-4 can also be applied in Kenya. Our Kenyan partner *icipe* translated and back-translated the PSS-4 into Kiswahili and English (see S2 Table for the final translation of the PSS-4). We analyzed the PSS-4 data as two distinct psychological factors: The psychological factor “perceived coping” consists of items 2 and 3 of the PSS-4 and measures the perceived ability to cope with stress. The other factor, consisting of items 1 and 4, asks about the amount of stress the respondent is experiencing and is referred to as “perceived stress”. Higher sum scores for the perceived stress factor indicate higher stress and higher sum scores for the perceived coping factor indicate a lower perceived ability to cope with stress. With regard to internal consistency in our data set, Cronbach's alpha for the one-factor model was less than 0.6 at 11 out of 12 measurement time points. For the two-factor model, the perceived stress factor had a Cronbach's alpha greater than 0.6 at eight out of the 12 time points and the perceived coping factor had a Cronbach's alpha greater than 0.6 at nine out of the 12 time points. Additionally, we analyzed the psychometric properties of the PSS-4 using a principal component analysis (PCA) for each measurement time point. The one-factor model explained a maximum of 47% of the variance while the two-factor model explained over 66% of the variance for each measurement time point (S3 and S4 Tables), thus supporting our choice of the two-factor model.

**Data collection method**

Throughout the RCT, the smallholder farmers received monthly SMS-based mobile phone surveys asking about their food security status [33]. Within these monthly surveys, the farmers were also asked whether someone in their household was currently pregnant. Farmers who answered “yes” to this question received an additional SMS-based mobile phone survey containing the PSS-4 questionnaire. Pregnant women could fill out the PSS-4 questionnaire via SMS survey every month while they were still pregnant. As soon as farmers answered “no” to the pregnancy question, they were excluded from the subsequent PSS-4 survey rounds. The observation period for this analysis was monthly from April 2020 until February 2021. The PSS-4 was seen as most suitable for use in SMS surveys as longer versions of the PSS are inefficient for repeated SMS surveys in larger populations in a developing country context [36]. For each completed survey round, participants received telephone credit (airtime) valued at 10 Kenyan Shilling. The treatment and control group received the same amount of airtime.

**Statistical analysis**

Intention-to-treat (ITT) analyses was used to analyze treatment effects (allocation and use of hermetic storage bags in the household) on perceived stress and perceived coping in pregnant women from smallholder farming households. ITT analysis is a recommended approach in RCTs when compliance and adherence to interventions cannot be guaranteed [58,59]. The ITT was estimated as the weighted average of the mean differences within pairs between treatment and control clusters [60] of pregnant women. For this purpose, arithmetic weights (wk) = n1k + n2k was used, representing the sum of the n observations in both clusters of each pair indexed by k. For further information see Imai and colleagues [60]. In addition, linear mixed-effects models was estimated for the entire observation period for both outcome measures, perceived stress and perceived coping, with the dichotomous variable treatment (yes/no) as the fixed effect and a random intercept that varied across the paired IDs (PID). Analyses were conducted using MATLAB and the open-source software R version 4.1.0 (the R Foundation for Statistical Computing, Vienna, Austria).

**Ethics approval**

Ethics approval was granted by the ETH Zurich Ethics Commission (EK, 2018-N-51) and *icipe’s* Science Committee (no approval number used). Registration of the study design can be found in the American Economic Association (AEA) RCT Registry [35]. Informed written consent of the households to participate in the study was obtained before the start of the intervention.

**Results**

**Sample characteristics**

During the observation period of April 2020 to February 2021, a total of 1,591 pregnant women from smallholder households participated in the SMS survey. Sample characteristics of baseline variables can be found in S5 Table. Baseline variables did not differ between control and treatment households, with the exception of slight differences in household size and age of household head. The households of the control group were slightly larger (M = 6.66, SD = 2.52) compared to those of the treatment group (M = 6.4, SD = 2.35) and the household heads of the control group (M = 45.21, SD = 12.78) were slightly older than the household heads of the treatment group (M = 44.14, SD = 12.35). However, these differences in household size (0.26) and age of household head (1.07 years) were very small.

**Perceived stress and perceived coping in pregnant women from control households**

In pregnant women in the control households, perceived coping scores were relatively stable during the lean season (between *M* = 4.4 and *M* = 4.6). After the end of the main harvest of 2020, perceived coping improved (lower mean values) for nearly all consecutive months (Fig 1). Perceived stress levels in pregnant women in the control households fluctuated over 12 consecutive months and were, overall, lower during the lean season. Compared to the lean season, pregnant women in the control households generally felt more stressed after the main harvest (Fig 2).

**Fig 1. Mean perceived coping scores and standard error for pregnant women in treatment and control households.**

Mean perceived coping is measured by items 2 and 3 of the PSS-4, with a 30-day recall period for all survey months. The weighted mean of all observations per survey month was used to measure the perceived coping factor (see statistical analysis section). Lower mean values indicate higher perceived coping. Vertical bars represent standard errors.

**Fig 2. Mean perceived stress scores and standard errors for pregnant women in treatment and control households.**

Mean perceived stress scores are measured by items 1 and 4 of the PSS-4, with a 30-day recall period for all survey months. The weighted mean of all observations per survey month was used to measure perceived stress scores (see statistical analysis section). Higher mean values indicate higher perceived stress. Vertical bars represent standard errors.

**Impact of improved on-farm storage on perceived coping in pregnant women**

For perceived stress and perceived coping, we expected the strongest treatment effects during the lean season when food stocks were depleted, which would then decrease after the start of the new storage period (the end of the main harvest). The results of the linear mixed-effects model show that pregnant women in the treatment households scored on average 0.167 points less than the control households on the coping measure over the entire observation period combined, which indicates higher perceived coping (*t*(6082) = -3.179, *p* = 0.001).

When analyzing each survey month separately, pregnant women in the treatment households perceived a higher ability to cope with stress in 11 out of 12 months compared to pregnant women in the control households (Fig 1). Perceived coping in pregnant women in the treatment households was significantly better during the lean season month of May 2020 (*p* = 0.014) and marginally better during the month of June 2020 (*p* = 0.094) (Fig 3). The difference between pregnant women in the treatment and control households was most substantial in September, right before the end of the main harvest of 2020 and the start of the new storage period, when pregnant women in the treatment households perceived themselves to cope significantly better with stress (*p* = 0.002). After the main harvest, pregnant women in the treatment households mostly continued to cope better with stress relative to the control group, although this difference was only significant in December 2020 (*p* = 0.005) (Fig 3). Supplementary S6 Table shows the ITT analysis for all survey months.

**Fig 3. Intention-to-treat (ITT) effects on perceived coping in pregnant women in smallholder farming households.**

For each survey month, the points represent the estimated ITT effects on the mean perceived coping scores. Vertical bars show 95% bootstrapped confidence intervals.

**Impact of improved on-farm storage on perceived stress in pregnant women**

Pregnant women in the treatment households scored an average of 0.13 points higher than those in the control households on the stress measure over the entire observation period combined, which indicates higher perceived stress (*t*(6082) = 2.314, *p* = 0.021).

When analyzing each survey month separately, pregnant women in the treatment households showed relatively stable perceived stress levels during the lean season, albeit higher compared to those in the control households (Fig 2). This difference was significant for the lean season months of May 2020 (*p* = 0.019), June 2020 (*p* = 0.004), August 2020 (*p* = 0.007) and September 2020 (*p* = 0.037) (Fig 4 and S7 Table). After the end of the main harvest in 2020, pregnant women in the treatment households more often felt less stressed than those in the control group, but this difference was only marginally significant for the month of January 2021 (*p* = 0.086).

**Fig 4. Intention-to-treat (ITT) effects on perceived stress in pregnant women from smallholder farming households.**

For each survey round, the points represent the estimated ITT effects on mean perceived stress scores. Vertical bars show 95% bootstrapped confidence intervals.

**Discussion**

The present study is the first RCT to analyze the causal effect of a food security intervention on perceived stress and perceived coping (component of overall mental health) in pregnant women from smallholder farming households in SSA. Our results show that pregnant women from the treatment households had higher perceived coping scores but experienced more perceived stress compared to pregnant women from the control households.

The implementation of the hermetic storage bags in the treatment households might have been evaluated as an externally determined environmental stressor that was uncertain, uncontrollable and threatening (primary appraisal), thus triggering a stress response. According to the results of the PSS-4 subscale perceived stress, pregnant women from the treatment households felt less able to control important things in their life and felt that difficulties were piling up so high that they could not overcome them (items 1 and 4) [36]. Previous research has demonstrated that participation in a new economic activity can initially increase psychological distress (measured using the PSS-10 as one factor) [61]. Hence, a lack of experience with this new on-farm storage technology, and uncertainty as to whether and how well these bags would work, may have contributed to the higher perceived stress levels in the treatment households. As individuals constantly interact with the environment, their cognitive processes (based on past experiences) can change. Therefore, after completion of the first harvest season and having gained the knowledge that these bags reduce food insecurity (adjusted primary appraisal), the significant effects of the treatment on perceived stress in pregnant women diminished and pregnant women in the treatment households therefore tended to be less stressed, albeit not significantly so. Moreover, the uptake of a new agricultural technology that targets food security may take longer to develop a positive impact on perceived stress. Stevenson et al. [62] measured how the implementation of an intervention to improve water quality for Ethiopian households affected psychological distress in Ethiopian women, and found no significant effect on psychological distress measured with the WHO Self-Reporting Questionnaire (SRQ-20). As a possible explanation for this finding, the authors suggested that the time between the implementation of the intervention and the stress measurement was too short to have yielded a potential treatment effect. Further research should implement longitudinal panel studies over several years to observe any effects.

Although the implementation of the new technology may pose a perceived threat, the technology itself is a tangible resource (secondary appraisal) that can be used to counteract the effects of PHL and therefore food insecurity. According to the subscale perceived coping of the PSS-4 [36]*,* pregnant women in the treatment households felt more confident about their ability to handle personal problems and felt more that things were going their way (items 2 and 3) compared to pregnant women in the control households. In SSA, women are generally in charge of feeding the household. This forces them to actively overcome food insecurity by adopting different coping strategies such as stretching food resources and skipping meals [63,64]. Given the higher perceived coping in pregnant women in the treatment households, one might assume that the intervention to reduce PHL empowered these women and gave them a tangible resource, as fewer food coping strategies were required [33]. Empowerment refers to “the process by which those who have been denied the ability to make choices acquire such an ability” [65]. Being deprived of basic needs reduces the ability to make meaningful choices [65]. Accordingly, it seems likely that pregnant women in the control households were deprived of choice over how to successfully feed their families, and this lack of empowerment attributable to food deprivation might have resulted in poorer perceived coping. This finding supports the social causation hypothesis that unfavorable environmental and economic conditions increase the risk of impaired mental health [66,67].

Mental health in pregnant women is affected by the time of gestation. Recent evidence indicates that the longitudinal course of distress during pregnancy exhibits a U-shaped curve [68], leading to the suggestion that the time of gestation might have impacted the effects of the treatment on perceived stress in our study. However, in contrast to observational studies, our study design reduces the risk of omitted variable bias as the matched-pair design rules out the influence of other potential confounding factors given that potential confounding factors should be similarly distributed in treatment and control households.

Some limitations need to be acknowledged when interpreting the findings reported in this study. First, the questionnaire was completed via self-report. However, as there was no personal interaction with the pregnant women, we do not expect a systematic response bias [69]. Second, we cannot guarantee that the pregnant women answered the SMS-based surveys themselves, although to minimize this potential problem we requested in our survey that the PSS-4 should be answered by the pregnant women. Third, it may have been preferable to use a stress questionnaire that better represents the cultural background of the study population, since perceived stress in the PSS-4 might not adequately reflect the local understanding of stress, namely “thinking too much” [70–73].

**Conclusion**

Our study is the first to examine the effect of an improved on-farm storage intervention on perceived stress and perceived coping in pregnant women from smallholder farming households in SSA. Our assumption that an intervention that mitigates food insecurity positively affects a component of maternal mental health was partially supported. In addition to studying the direct impact of an improved on-farm storage intervention on perceived stress and coping in pregnant women, future research should focus on how this storage technology impacts maternal mental health in a broader sense and whether biological mechanisms, such as epigenetics, may underlie this association. Such findings could have important implications for countries in SSA, where women in rural areas are strongly affected by food insecurity and mental health problems [74], and could contribute to the achievement of the second and third goals of the United Nations Agenda for Sustainable Development [75], namely to end food insecurity and ensure well-being and mental health.

**Acknowledgements**

We thank the International Centre of Insect Physiology and Ecology (*icipe*) for their great collaboration and excellent support in the field.

**References**

1.	Stillerman KP, Mattison DR, Giudice LC, Woodruff TJ. Environmental exposures and adverse pregnancy outcomes: A review of the science. Reprod Sci. 2008;15: 631–650. doi:10.1177/1933719108322436

2.	Ivers LC, Cullen KA. Food insecurity: special considerations for women. Am J Clin Nutr. 2011;94: 1740S-1744S. doi:10.3945/ajcn.111.012617

3.	Van den Bergh BRH, van den Heuvel MI, Lahti M, Braeken M, de Rooij SR, Entringer S, et al. Prenatal developmental origins of behavior and mental health: The influence of maternal stress in pregnancy. Neurosci Biobehav Rev. 2020;117: 26–64. doi:10.1016/j.neubiorev.2017.07.003

4.	Lindsay KL, Gibney ER, McAuliffe FM. Maternal nutrition among women from sub‐Saharan Africa, with a focus on Nigeria, and potential implications for pregnancy outcomes among immigrant populations in developed countries. J Hum Nutr Diet. 2012;25: 534–546. doi:10.1111/j.1365-277X.2012.01253.x

5.	Koletzko B, Godfrey KM, Poston L, Szajewska H, Van Goudoever JB, De Waard M, et al. Nutrition during pregnancy, lactation and early childhood and its implications for maternal and long-term child health: The early nutrition project recommendations. Ann Nutr Metab. 2019;74: 93–106. doi:10.1159/000496471

6.	Cox JT, Phelan ST. Nutrition during pregnancy. Obstet Gynecol Clin North Am. 2008;35: 369–383. doi:10.1016/j.ogc.2008.04.001

7.	Lartey A. Maternal and child nutrition in Sub-Saharan Africa: challenges and interventions. Proc Nutr Soc. 2008;67: 105–108. doi:10.1017/S0029665108006083

8.	Iqbal S, Ali I. Maternal food insecurity in low-income countries: Revisiting its causes and consequences for maternal and neonatal health. J Agric Food Res. 2021;3: 100091. doi:10.1016/j.jafr.2020.100091

9.	Cheu LA, Yee LM, Kominiarek MA. Food insecurity during pregnancy and gestational weight gain. Am J Obstet Gynecol MFM. 2020;2. doi:10.1016/j.ajogmf.2019.100068

10.	Lazarus RS, Folkman S. Stress, appraisal, and coping. Springer Publishing Company; 1984.

11.	Folkman S. Personal control and stress and coping processes: a theoretical analysis. J Pers Soc Psychol. 1984;46: 839–852. doi:10.1037//0022-3514.46.4.839

12.	Lazarus R. Psychological stress and coping process. New York: McGraw-Hill; 1966.

13.	Biggs A, Brough P, Drummond S. Lazarus and Folkman’s psychological stress and coping theory. The handbook of stress and health: a guide to research and practice. 2017;351–364. doi:10.1002/9781118993811.ch21

14.	Trudell JP, Burnet ML, Ziegler BR, Luginaah I. The impact of food insecurity on mental health in Africa: A systematic review. Soc Sci Med. 2021;278: 113953. doi:10.1016/j.socscimed.2021.113953

15.	Jebena MG, Taha M, Nakajima M, Lemieux A, Lemessa F, Hoffman R, et al. Household food insecurity and mental distress among pregnant women in Southwestern Ethiopia: A cross sectional study design. BMC Pregnancy Childbirth. 2015;15: 1–9. doi:10.1186/s12884-015-0699-5

16.	Van Praag HM. Can stress cause depression? Prog Neuro-Psychopharmacology Biol Psychiatry. 2004;28: 891–907. doi:10.1016/j.pnpbp.2004.05.031

17.	Hammen C. Stress and depression. Annu Rev Clin Psychol. 2005;1: 293–319. doi:10.1146/annurev.clinpsy.1.102803.143938

18.	Yang L, Zhao Y, Wang Y, Liu L, Zhang X, Li B, et al. The Effects of Psychological Stress on Depression. Curr Neuropharmacol. 2015;13: 494–504. doi:10.2174/1570159x1304150831150507

19.	Abrahams Z, Lund C, Field S, Honikman S. Factors associated with household food insecurity and depression in pregnant South African women from a low socio-economic setting: a cross-sectional study. Soc Psychiatry Psychiatr Epidemiol. 2018;53: 363–372. doi:10.1007/s00127-018-1497-y

20.	Dibaba Y, Fantahun M, Hindin MJ. The association of unwanted pregnancy and social support with depressive symptoms in pregnancy: evidence from rural Southwestern Ethiopia. BMC Pregnancy Childbirth. 2013;13: 135. doi:10.1186/1471-2393-13-135

21.	Ngure MW, Wandiga SO, Olago DO, Oriaso SO. Climate change stressors affecting household food security among Kimandi-Wanyaga smallholder farmers in Murang’a County, Kenya. Open Agric. 2021;6: 587–608. doi:10.1515/opag-2021-0042

22.	Gebre GG, Rahut DB. Prevalence of household food insecurity in East Africa: Linking food access with climate vulnerability. Clim Risk Manag. 2021;33: 100333. doi:10.1016/j.crm.2021.100333

23.	Kotir JH. Climate change and variability in Sub-Saharan Africa: A review of current and future trends and impacts on agriculture and food security. Environ Dev Sustain. 2011;13: 587–605. doi:10.1007/s10668-010-9278-0

24.	Kaminski J, Christiaensen L, Gilbert CL. Seasonality in local food markets and consumption: Evidence from Tanzania. Oxf Econ Pap. 2016;68: 736–757. doi:10.1093/oep/gpw013

25.	Shukla S, Husak G, Turner W, Davenport F, Funk C, Harrison L, et al. A slow rainy season onset is a reliable harbinger of drought in most food insecure regions in Sub-Saharan Africa. PLoS One. 2021;16: e0242883. doi:10.1371/journal.pone.0242883

26.	Stathers T, Lamboll R, Mvumi BM. Postharvest agriculture in changing climates: Its importance to African smallholder farmers. Food Secur. 2013;5: 361–392. doi:10.1007/s12571-013-0262-z

27.	Hodges RJ, Buzby JC, Bennett B. Postharvest losses and waste in developed and less developed countries: Opportunities to improve resource use. J Agric Sci. 2011;149: 37–45. doi:10.1017/S0021859610000936

28.	Tefera T. Post-harvest losses in African maize in the face of increasing food shortage. Food Secur. 2012;4: 267–277. doi:10.1007/s12571-012-0182-3

29.	Chegere MJ. Post-harvest losses reduction by small-scale maize farmers: The role of handling practices. Food Policy. 2018;77: 103–115. doi:10.1016/j.foodpol.2018.05.001

30.	Affognon H, Mutungi C, Sanginga P, Borgemeister C. Unpacking postharvest losses in sub-Saharan Africa: A Meta-Analysis. World Dev. 2015;66: 49–68. doi:10.1016/j.worlddev.2014.08.002

31.	Bonuedi I, Kornher L, Gerber N. Agricultural seasonality, market access, and food security in Sierra Leone. Food Secur. 2022;14: 471–494. doi:10.1007/s12571-021-01242-z

32.	Brander M, Bernauer T, Huss M. Improved on-farm storage reduces seasonal food insecurity of smallholder farmer households – Evidence from a randomized control trial in Tanzania. Food Policy. 2020; 101891. doi:10.1016/j.foodpol.2020.101891

33.	Huss M, Brander M, Kassie M, Ehlert U, Bernauer T. Improved storage mitigates vulnerability to food-supply shocks in smallholder agriculture during the COVID-19 pandemic. Glob Food Sec. 2021;28: 100468. doi:10.1016/j.gfs.2020.100468

34.	Bruhn BM, Mckenzie D. In pursuit of balance : Randomization in practice in development field experiments. Am Econ J : Appl Econ. 2009;1: 200–232. doi:10.1257/app.1.4.200

35.	Huss M, Brander M, Kassie M, Ehlert U, Bernauer T. Welfare, nutritional, and human health Impacts of post-harvest loss prevention: a large-scale field Experiment in Kenya (IMPACT (AEA RCT Registry). 2020. Available from: https://doi.org/10.1257/rct .5845-1.1

36.	Cohen S, Kamarck T, Mermelstein R. A global measure of perceived stress. J Health Soc Behav. 1983;24: 385–396. doi: 10.2307/2136404

37.	Cohen S, Wiliamson GM. Perceived stress in a probability sample of the United States. In: Spacapan S, Oskamp S, editors. The Social Psychology of Health. Newbury Park, CA: Sage Publications; 1988. pp. 31–67.

38.	Taylor JM. Psychometric analysis of the ten-item perceived stress scale. Psychol Assess. 2015;27: 90–101. doi:10.1037/a0038100

39.	Andreou E, Alexopoulos EC, Lionis C, Varvogli L, Gnardellis C, Chrousos GP, et al. Perceived stress scale: reliability and validity study in Greece. Int J Environ Res Public Health. 2011;8: 3287–3298. doi:10.3390/ijerph8083287

40.	Lee EH. Review of the psychometric evidence of the perceived stress scale. Asian Nurs Res. 2012;6: 121–127. doi:10.1016/j.anr.2012.08.004

41.	Leung DY, Lam TH, Chan SS. Three versions of perceived stress scale: Validation in a sample of Chinese cardiac patients who smoke. BMC Public Health. 2010;10. doi:10.1186/1471-2458-10-513

42.	Figalová N, Charvát M. The perceived stress scale: reliability and validity study in the Czech Republic. Cesk Psychol. 2021;65: 46–59. doi:10.51561/cspsych.65.1.46

43.	Kim HJ. Reliability and validity of the 4-Item version of the Korean Perceived Stress Scale. Res Nurs Heal. 2016;39: 472–479. doi:10.1002/nur.21745

44.	Perera MJ, Brintz CE, Birnbaum-Weitzman O, Penedo FJ, Gallo LC, Gonzalez P, et al. Factor structure of the perceived stress scale-10 (PSS) across English and Spanish language responders in the HCHS/SOL sociocultural ancillary study. Psychol Assess. 2017;29: 320–328. doi:10.1037/pas0000336

45.	Michaelides MP, Christodoulou A, Kkeli N, Karekla M, Panayiotou G. Factorial structure of the perceived stress scale and implications for scoring. Eur Rev Appl Psychol. 2016;66: 309–316. doi:10.1016/j.erap.2016.07.002

46.	Ruisoto P, López-Guerra VM, Paladines MB, Vaca SL, Cacho R. Psychometric properties of the three versions of the Perceived Stress Scale in Ecuador. Physiol Behav. 2020;224: 113045. doi:10.1016/j.physbeh.2020.113045

47.	Vallejo MA, Vallejo-Slocker L, Fernández-Abascal EG, Mañanes G. Determining factors for stress perception assessed with the Perceived Stress Scale (PSS-4) in Spanish and other European samples. Front Psychol. 2018;9. doi:10.3389/fpsyg.2018.00037

48.	Yokokura AVCP, da Silva AAM, de Kássia Braga Fernandes J, Del-Ben CM, de Figueiredo FP, Barbieri MA, et al. Perceived Stress Scale: confirmatory factor analysis of the PSS14 and PSS10 versions in two samples of pregnant women from the BRISA cohort. Cad Saude Publica. 2017;33: 1–13. doi:10.1590/0102-311X00184615

49.	Solivan AE, Wallace ME, Kaplan KC, Harville EW. Use of resiliency framework to examine pregnancy and birth outcomes among adolescents : A qualitative study. Fam Syst Health. 2016;33: 349–355. doi:10.1037/fsh0000141

50.	Chaaya M, Osman H, Naassan G, Mahfoud Z. Validation of the Arabic version of the Cohen perceived stress scale (PSS-10) among pregnant and postpartum women. BMC Psychiatry. 2010;10. doi:10.1186/1471-244X-10-111

51.	Musana JW, Cohen CR, Kuppermann M, Gerona R, Wanyoro A, Aguilar D, et al. Association of differential symptoms of stress to hair cortisol and cortisone concentrations among pregnant women in Kenya. Stress. 2020;23: 556–566. doi:10.1080/10253890.2019.1696305

52.	Samia P, Premji S, Tavangar F, Yim IS, Wanyonyi S, Merali M, et al. Adverse childhood experiences and changing levels of psychosocial distress scores across pregnancy in Kenyan women. Int J Environ Res Public Health. 2020;17. doi:10.3390/ijerph17103401

53.	Ice GH, Sadruddin AFA, Vagedes A, Yogo J, Juma E. Stress associated with caregiving: An examination of the stress process model among Kenyan Luo elders. Soc Sci Med. 2012;74: 2020–2027. doi:10.1016/j.socscimed.2012.02.018

54.	Murnane PM, Miller JD, Tuthill EL, Collins SM, Neilands TB, Onono M, et al. Perinatal food insecurity and postpartum psychosocial stress are positively associated among Kenyan women of mixed HIV status. AIDS Behav. 2020;24: 1632–1642. doi:10.1007/s10461-019-02676-5

55.	Afulani PA, Ongeri L, Kinyua J, Temmerman M, Mendes WB, Weiss SJ. Psychological and physiological stress and burnout among maternity providers in a rural county in Kenya: individual and situational predictors. BMC Public Health. 2021;21: 1–16. doi:10.1186/s12889-021-10453-0

56.	Goodman ML, Gutarra C, Billingsley KM, Keiser PH, Gitari S. Childhood exposure to emotional abuse and later life stress among Kenyan women: a mediation analysis of cross-sectional data. Anxiety Stress Coping. 2017;30: 469–483. doi:10.1080/10615806.2016.1271876

57.	Shah SSNH, Laving A, Okech-Helu VC, Kumar M. Depression and its associated factors: perceived stress, social support, substance use and related sociodemographic risk factors in medical school residents in Nairobi, Kenya. BMC Psychiatry. 2021;21: 1–15. doi:10.1186/s12888-021-03439-0

58.	Gupta S. Intention-to-treat concept: A review. Perspect Clin Res. 2011;2: 109. doi:10.4103/2229-3485.83221

59.	McCoy CE. Understanding the intention-to-treat principle in randomized controlled trials. West J Emerg Med. 2017;18: 1075–1078. doi:10.5811/westjem.2017.8.35985

60.	Imai K, King G, Nall C. The essential role of pair matching in cluster-randomized experiments, with application to the Mexican Universal Health Insurance Evaluation. Stat Sci. 2009;24: 29–53. doi:10.1214/08-STS274

61.	Fernald LCH, Hamad R, Karlan D, Ozer EJ, Zinman J. Small individual loans and mental health: A randomized controlled trial among South African adults. BMC Public Health. 2008;8: 1–14. doi:10.1186/1471-2458-8-409

62.	Stevenson EGJ, Ambelu A, Caruso BA, Tesfaye Y, Freeman MC. Community water improvement, household water insecurity, and women’s psychological distress : an intervention and control study in Ethiopia. PloS one. 2016; 11: e0153432. doi:10.5061/dryad.9824c

63.	Hadley C, Stevenson EGJ, Tadesse Y, Belachew T. Rapidly rising food prices and the experience of food insecurity in urban Ethiopia: impacts on health and well-being. Soc Sci Med. 2012;75: 2412–2419. doi:10.1016/j.socscimed.2012.09.018

64.	Piperata BA, Salazar M, Schmeer KK, Herrera Rodríguez A. Tranquility is a child with a full belly: Pathways linking food insecurity and maternal mental distress in Nicaragua. Ecol Food Nutr. 2020;59: 79–103. doi:10.1080/03670244.2019.1671835

65.	Kabeer N. Resources, agency, achievements: Reflections on the measurement of women’s empowerment. Dev Change. 1999;30: 435–464.

66.	Dohrenwend BP, Levav I, Shrout PE, Schwartz S, Naveh G, Link BG, et al. Socioeconomic status and psychiatric disorders: The causation-selection issue. Science. 1992;255: 946–952. doi:10.1126/science.1546291

67.	Hudson CG. Socioeconomic status and mental illness: tests of the social causation and selection hypotheses. Amer J Orthopsychiatry. 2005;75: 3–18. doi: 10.1037/0002-9432.75.1.3

68.	Markon KE, Brunette CA, Whitney BM, O’Hara MW. Mood during pregnancy: trends, structure, and invariance by gestational day. J Psychiatr Res. 2021;140: 260–266. doi:10.1016/j.jpsychires.2021.06.006

69.	Krumpal I. Determinants of social desirability bias in sensitive surveys: A literature review. Qual Quant. 2013;47: 2025–2047. doi:10.1007/s11135-011-9640-9

70.	Ice GH, Yogo J. Measuring Stress Among Luo Elders: Development of the Luo Perceived Stress Scale. Field methods. 2005;17: 394–411. doi:10.1177/1525822X05280176

71.	Patel V. Cultural factors and international epidemiology. Br Med Bull. 2001; 33–45. doi:10.1177/001440296903500608

72.	Kaiser BN, Haroz EE, Kohrt BA, Bolton PA, Bass JK, Hinton DE. “Thinking too much”: A systematic review of a common idiom of distress. Soc Sci Med. 2015;147: 170–183. doi:10.1016/j.socscimed.2015.10.044

73.	Pienkowski T, Keane A, Kinyanda E, Knizek BL, Muhanguzi G, Medicine T. The role of nature conservation and commercial farming in psychological distress among rural Ugandans. bioRxiv. [Preprint]. 2021. [posted 2021 June 09]. Available from: https://www.biorxiv.org/content/10.1101/2021.06.08.446718v1 doi:10.1101/2021.06.08.446718

74.	Sinclair K, Ahmadigheidari D, Dallmann D, Miller M, Melgar-Quiñonez H. Rural women: most likely to experience food insecurity and poor health in low- and middle-income countries. Glob Food Sec. 2019;23: 104–115. doi:10.1016/j.gfs.2019.04.006

75.	United Nations. Transforming our world: the 2030 agenda for sustainable development. United Nations. 2015. Available from: https://sustainabledevelopment.un.org/content/documents/21252030%20Agenda%20for%20Sustainable%20Development%20web.pdf

**Supporting information**

**S1 Fig. Study region and location of smallholder farmers divided into treatment and control group.**

**S2 Table. Translation of the short form of the Perceived Stress Scale (PSS-4).**

**S3 Table. Cumulative variances of principal component analysis (PCA) for one-factor model and two-factor model.**

**S4 Table. Minimum and maximum factor loadings for one-factor model and two-factor model.**

**S5 Table. Sample characteristics of baseline variables between treatment and control group.**

**S6 Table. Effects of improved on-farm storage on stress coping abilities during pregnancy.**

**S7 Table. Effects of improved on-farm storage on perceived stress during pregnancy.**
